# Development of spray-drying-based surface-enhanced Raman spectroscopy

**DOI:** 10.1038/s41598-022-08598-y

**Published:** 2022-03-16

**Authors:** Chigusa Matsumoto, Masao Gen, Atsushi Matsuki, Takafumi Seto

**Affiliations:** 1 Graduate School of Science and Technology, Kanazawa University, Kanazawa, 920-1192 Japan; 2 Faculty of Frontier Engineering, Institute of Science and Engineering, Kanazawa University, Kanazawa, 920-1192 Japan; 3 Institute of Nature and Environmental Technology, Kanazawa University, Kanazawa, 920-1192 Japan

**Keywords:** Environmental sciences, Chemical engineering

## Abstract

We report a spray-drying method to fabricate silver nanoparticle (AgNP) aggregates for application in surface-enhanced Raman spectroscopy (SERS). A custom-built system was used to fabricate AgNP aggregates of four sizes, 48, 86, 151, and 218 nm, from drying droplets containing AgNPs atomized from an AgNP suspension. Sample solutions of Rhodamine B (RhB) at 10^–6^, 10^–8^, and 10^–10^ M concentrations were dropped onto the AgNP aggregates as probe molecules to examine the enhancement of the Raman signals of the RhB. The ordering of the analytical enhancement factors (AEFs) by aggregate size at a 10^–6^ M RhB was 86 nm > 218 nm > 151 nm > 48 nm. When RhB concentrations are below 10^–8^ M, the 86 and 151 nm AgNP aggregates show clear RhB peaks. The AEFs of the 86 nm AgNP aggregates were the highest in all four aggregates and higher than those of the 218-nm aggregates, although the 218-nm aggregates had more hot spots where Raman enhancement occurred. This finding was attributable to the deformation and damping of the electron cloud in the highly aggregated AgNPs, reducing the sensitivity for Raman enhancement. When RhB was premixed with the AgNP suspension prior to atomization, the AEFs at 10^–8^ M RhB rose ~ 100-fold compared to those in the earlier experiments (the post-dropping route). This significant enhancement was probably caused by the increased opportunity for the trapping of the probe molecules in the hot spots.

## Introduction

Water contamination is a global concern that threatens human health^1–4^ and impairs the health of ecosystems^5–7^. Water contaminants and pollutants include heavy metals^8^ and organic compounds that adversely affect human health and ecosystems^9–12^. Water contamination needs to be promptly detected and analyzed to sustain the environment and minimize human health hazards and ecosystem health impairments.

Heavy metal in water samples is generally detected by methods such as atomic absorption spectrometry (AAS)^13,14^, anodic stripping voltammetry (ASV)^15^, inductively coupled plasma mass spectrometry (ICP-MS)^16,17^ and electrochemical detection^18,19^. HPLC/ICP-MS coupling detects heavy metals at rapid speeds in water even at concentrations on the order of ppt^20^, while the electrochemical methods are capable of detecting heavy metals onsite^21^. Organic pollutants, on the other hand, are detected by chromatographic and spectroscopic methods^22,23^. The multiple procedures required to prepare the samples for these methods introduce a risk of adulteration of the samples with impurities. Impurities may enter the samples, for example, in pretreatment steps such as the liquid–liquid extraction and solid-phase extraction^24^. Volatile components, moreover, are likely to be lost during the solid-phase extraction^25^.

Surface-enhanced Raman spectroscopy (SERS) is a facile and rapid platform for the trace analysis of organic compounds that provides a drastic enhancement of the Raman signals over traditional Raman spectroscopy. SERS stems from localized surface plasmon resonances in noble metal nanoparticles that create enormously intensified electromagnetic fields^26^. The significant Raman enhancement in SERS occurs at the junctions of two or more aggregated metal nanoparticles^27–30^, so-called hot spots. The degree of aggregation of the noble metal nanoparticles is a critical determinant of Raman enhancement in SERS. Hence, a number of studies have been conducted to control these aggregates^31,32^. The placement of probe molecules in the hot spots is also an essential step to enhance their Raman signals^33^. When gas adsorption on noble metal nanoparticles is facilitated, SERS can be used for gas analysis^34^.

**Figure 1:**
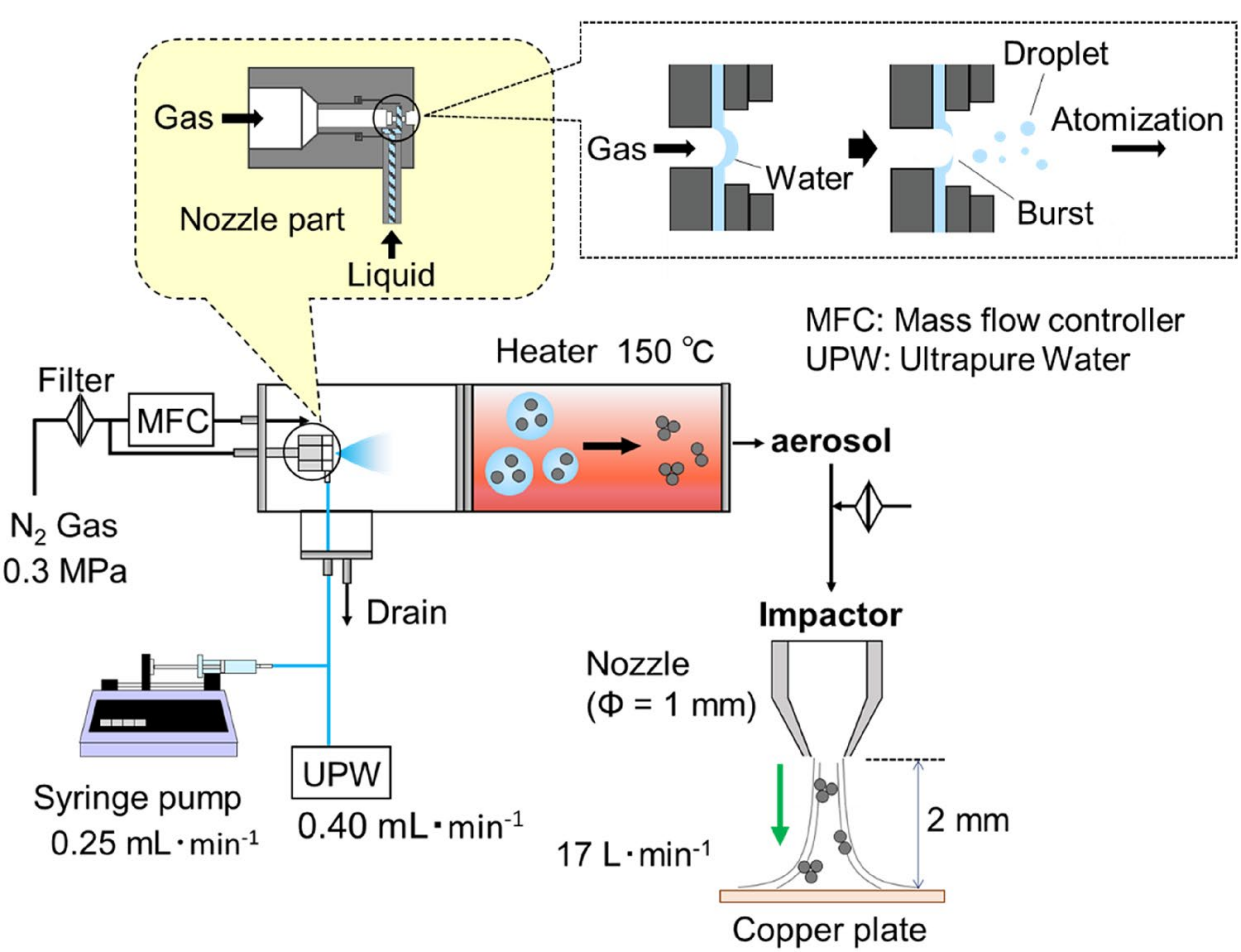
Schematic of an atomization system for the fabrication of aggregated silver nanoparticles.

Aerosol techniques can be flexibly applied to many research fields, from pollution studies to the detection of biological species^35^ and investigations into material synthesis. For instance, SERS using electrospray has been proposed for surface characterizations^36,37^. One such technique, the spray-drying method, atomizes tiny droplets from stock solutions. An investigator performing the spray-drying method can control the degree of nanoparticle aggregation by varying the droplet size and the concentration of nanoparticles within the droplet^38^. The nanoparticle aggregate is formed as a result of solvent evaporation inside the droplets (Fig. 1). The probe molecules can also get trapped in the hot spots during aggregate formation, which may provide a mechanism for arranging the probe molecules in the hot spots more efficiently than can be done by methods using traditional SERS substrates. In the SERS substrate method, for example, the probe molecules need to diffuse into the hot spots when the analyte solution is dropped onto the SERS substrate^39^. An alternative approach for performing SERS has been developed using a colloidal suspension^40,41^. The aggregation state of the nanoparticles, however, is commonly controlled with the use of additives, that is, surfactants. The Raman signals of these additives can also be enhanced, which can potentially interfere with the signals of the probe molecules. In contrast, a spray-drying method enables the creation of nanoparticle aggregates without surfactants.

In this study we report a spray-drying method to be applied for the SERS analysis of trace amounts of probe molecules. First, we demonstrate the controllable fabrication of nanoparticle aggregates using a custom-built spray-drying system. We examine how the size of silver nanoparticle aggregates affects the SERS signals using Rhodamine B as a probe molecule. Lastly, we atomize a premixed solution of silver nanoparticles and Rhodamine B to facilitate the trapping of the probe molecules in hot spots in order to further enhance their Raman signals.

## Results and discussion

### Size and morphology characterization

**Figure 2:**
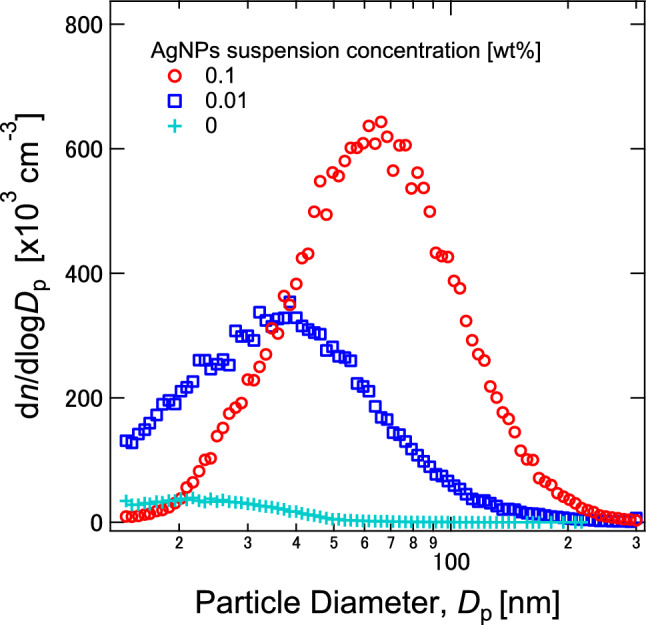
Size distributions of the AgNPs generated from the 0.01-, 0.1-, and 0-wt% sample suspensions. Note that the 0 wt% condition used only ultrapure water for the atomization.

Figure 2 shows the gas-phase size distributions of AgNP aerosols atomized from AgNP suspensions of 0.01 and 0.1 wt%. The average size of an AgNP aerosol from the 0.01-wt% suspension was found to be about 38 nm, which was roughly consistent with the size of a primary AgNP particle (~ 30 nm) in the suspension. This agreement in size confirmed that a droplet generated from the 0.01 wt% suspension mainly contained a single AgNP. The width of the distribution was reflected by the size distribution of the droplets generated in the atomization system. The average droplet diameter was estimated to be about 1.6 μm, based on the size of the AgNP aerosol shown in Text S1 in the Supplementary Information (SI). When the suspension concentration of 0.1 wt% was used to generate AgNP aerosols, the average size increased to 66 nm (Fig. 2), more than double the size measured when using the 0.01 wt% suspension. On the basis of the mass balance^42^, the tenfold higher concentration of the suspension resulted in a threefold larger particle size at a given droplet size and given particle density, assuming that a spherical aerosol was formed from the solvent evaporation of a droplet containing colloidal particle(s). Hence, the increased average size could be attributed to the increase in the concentration of AgNPs in the droplet, that is, the suspension concentration. This size increase also suggests that a droplet generated from the 0.1 wt% suspension contained more than one AgNP, leading to aggregate formation. Overall, the above results demonstrate that the sizes of the aerosols generated from our system were controllable by the suspension concentration.

**Figure 3:**
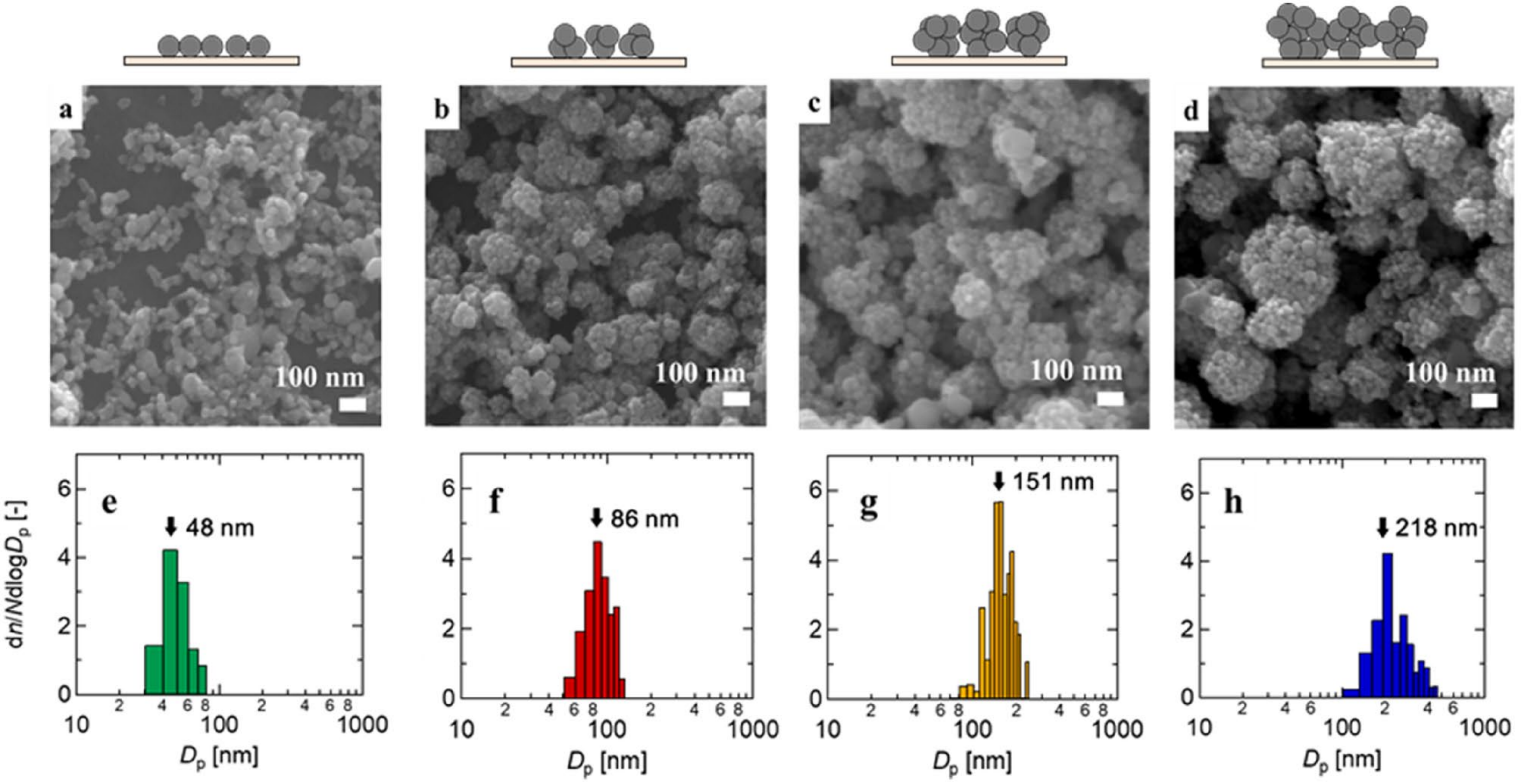
SEM images and size distributions of the deposited AgNPs. (**a, e**) 48 nm, (**b, f**) 86 nm, (**c, g**) 151 nm, and (**d, h**) 218 nm.

The SEM images in Fig. 3 exhibit AgNPs deposited on the copper plates without probe molecules (i.e., RhB) added, and the corresponding size distributions of the four AgNP aggregates. AgNPs generated from the 0.01 wt% suspension formed submonolayer films (Fig. 3a). The average measured size of the primary particles was 48 nm (Fig. 3e), which was in fair agreement with the primary particle size (30 nm) and aerosol particle size (38 nm). This agreement in size suggests that the AgNPs were deposited on the substrate without much aggregate formed in the air prior to impaction. The medium aggregate sizes were obtained by using a 0.1-wt% AgNP suspension to generate AgNP aerosols with a broad size range (Fig. 2) and then using a differential mobility analyzer (DMA) to extract only particles of around 70 or 120 nm. The deposited AgNPs formed spherical aggregates (Fig. 3b or c) with an average measured aggregate size of 86 or 151 nm (Fig. 3f or g). Without DMA for size selection, the deposited AgNPs generated from the 0.1-wt% suspension formed even larger spherical aggregates (Fig. 3d) with an aggregate size of 218 nm (Fig. 3h) more than double that of the AgNP aerosols found in the gas phase measurement (Fig. 2). This size difference between the gas phase measurement and SEM observation may have been attributable to the particle bounce effect^43^. As discussed in the Supplementary Information (Text S3), we expected particle bounce to occur for all of the deposited particles studied. As a result, the AgNPs deposited on the substrate may have been redistributed by this expected particle bounce. Only large aggregate AgNPs, which have a higher adhesion force due to the higher contact area between a particle and a substrate^44^, may remain deposited. Using the two suspension concentrations and DMA, we fabricated aggregates of four different sizes: 48, 86, 151 and 218 nm.

### Aggregate size dependence of Raman enhancement

**Figure 4:**
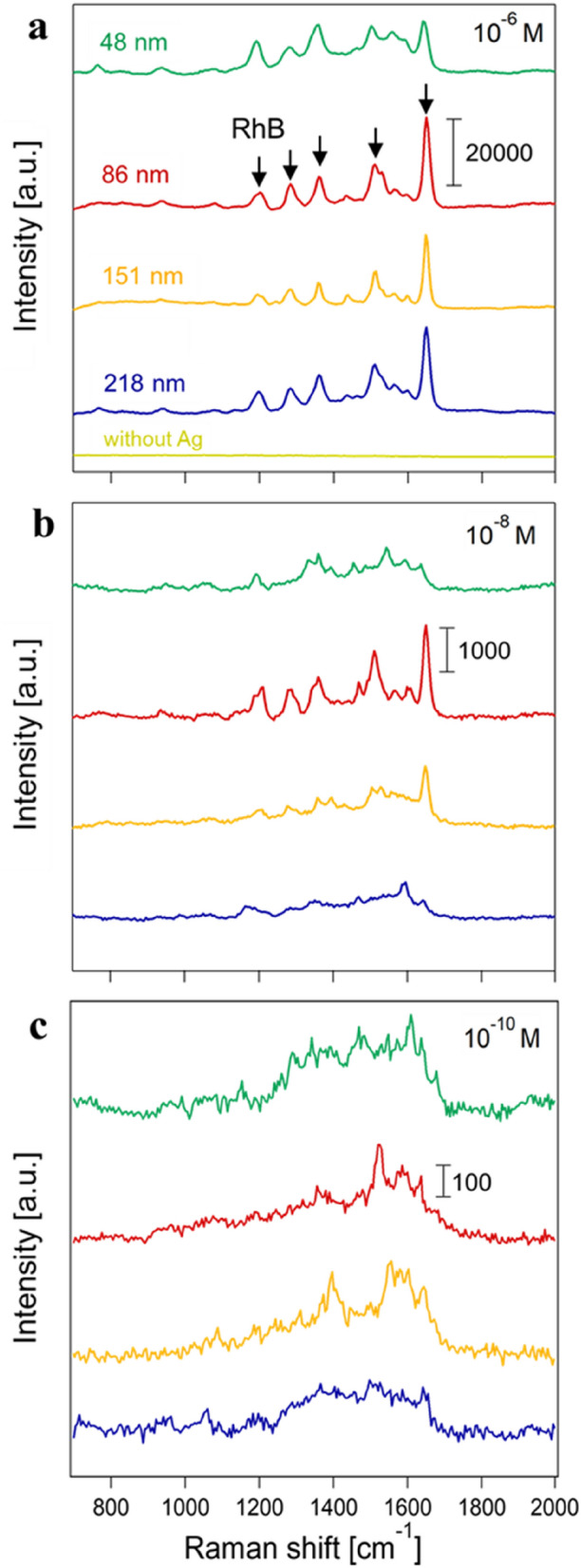
SERS spectra of RhB at the (**a**) 10^−6^ M, (**b**) 10^−8^ M, and (**c**) 10^−10^ M concentrations. The arrows indicate the representative RhB peaks (Table 1).

Figure 4 shows the SERS spectra of RhB at 10^−6^, 10^−8^, and 10^−10^ M using the aggregate sizes of 48, 86, 151, and 218 nm. A standard RhB solution was dropped onto the aggregates. At 10^−6^ M RhB, all of the characteristic vibrational peaks were clearly observed at 500–1700 cm^−1^ for the AgNP aggregates of all four sizes (Fig. 4a). The peak assignments are listed in Table 1^45^. Among the peaks, the C–C stretching mode at 1649 cm^−1^ was found to be most intense, and thus was used as the representative peak for calculating an enhancement factor. No peaks were seen in the absence of AgNPs (Fig. 4a), which confirms that the AgNP aggregates were responsible for the Raman enhancement of the RhB peaks. The present method was compared to the colloidal suspension method. In the colloidal suspension method, one hundred µL of 1.0 × 10^−6^, 1.0 × 10^−8^, or 1.0 × 10^−10^ M RhB solution was mixed with one hundred µL of 0.1 wt% AgNP aqueous suspension. Then, one hundred µL of the mixed solution was dropped on the copper substrate and the substrate was left to dry naturally. Totally 50 SERS spectra obtained at 10^−6^ M RhB show no clear peaks of RhB. (Fig. S1 in SI) The same is true for SERS spectra at 10^−8^ and 10^−10^ M RhB. The results suggest that AgNP aggregates produced by our method have higher sensitivity than the colloidal suspension method.

**Table 1:** Main peaks of Rhodamine B and the peak assignments.

Raman shift [cm^−1^]	Assignments
1199	C–C bridge band stretching and aromatic
1281	C–H bending
1360	Aromatic C–C bending
1528	C–H stretching
1649	C–C stretching vibration mode

Raman enhancement generally increases with the number of contact points (hot spots) between SERS nanoparticles of a given primary size^27–29^ in the laser sensing volume. Hence, the AgNPs of the largest aggregate size in the present study, 218 nm, were expected to show the highest peak intensity at 1649 cm^−1^, as they had the largest number of contact points. We were interested to find, however, that the medium-size AgNPs (86 nm) exhibited the highest target peak. By aggregate size, the peak intensity follows the order 86 nm > 218 nm > 151 nm > 48 nm. The peak intensity from the 218-nm aggregates was higher than that from the 48-nm aggregates, which can be explained by the larger number of contact points. The higher peak from the 86-nm aggregate versus that from the 218-nm aggregate was attributable to deformation and damping of the electron cloud in the high degree of AgNP aggregation^31,32^. The deformation and damping effect cause redshifts of the surface plasmon excitation wavelength of the AgNPs. The redshifts reduce the excitation, and therefore the sensitivity for Raman enhancement. Similar observations have been made on the AgNP aggregates in the size range from 520 to 1600 nm^39^, with a higher Raman enhancement observed for the smaller aggregates. At the 10^−8^ M RhB (Fig. 4b), the 86- and 151-nm aggregates alone clearly showed the RhB peaks, but no RhB peaks were seen for the 48 and 218 nm aggregates. The 48 and 218 nm aggregates exhibited some peaks which could not be assigned to RhB. These peaks were found to be non-reproducible and likely due to the photocarbonization^46,47^. No clear RhB peaks were seen in any of the aggregates at 10^–10^ M RhB (Fig. 4c), suggesting detection limits of > 10^–10^ M and > 10^–8^ M RhB for the 86 and 151 nm aggregates, and the 48 and 218 nm aggregates, respectively, in this study.

**Figure 5:**
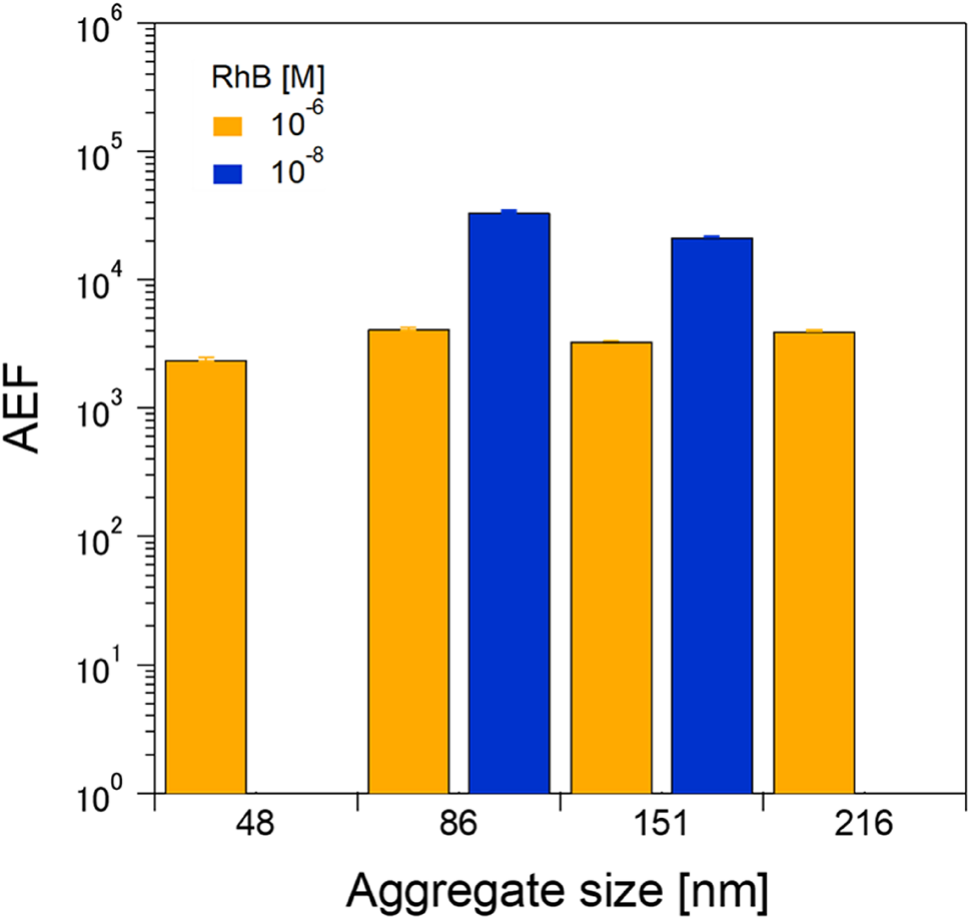
AEF values at 10^–6^ and 10^–8^ M RhB concentrations for the AgNP aggregate sizes of 48, 86, 151 nm, and 218 nm. No AEF values were calculated the 48- and 218-nm aggregate at the 10^–8^ M RhB concentrations because of the absence of RhB peaks.

The aggregate size dependence of Raman enhancement was summarized in terms of the analytical enhancement factor (AEF) for the RhB representative peak at ~ 1650 cm^−1^ (Fig. 5). The AEF was calculated with the following formula^48^

1 $$\mathrm{AEF}=\frac{{I}_{\mathrm{SERS}}/{N}_{\mathrm{SERS}}}{{I}_{\mathrm{RS}}/{N}_{\mathrm{RS}}}$$

where *I*_SERS_ is the intensity of RhB with a given aggregate size of AgNPs in the SERS experiments, and *I*_RS_ is the intensity of RhB without AgNPs (i.e., normal Raman measurement). *N*_SERS_ and *N*_RS_ are the numbers of RhB molecules for *I*_SERS_ and *I*_RS_, respectively, in the laser sensing volume. Note that we assume that *N*_SERS_ equals *N*_RS_ in this study, as the laser sensing volume is the same in the SERS and normal Raman measurements. The SERS experiments for 10^–6^ M RhB gave AEF values of 2.4 × 10^3^, 4.2 × 10^3^, 3.3 × 10^3^, and 4.0 × 10^3^ for the 48-, 86-, 151-, and 218-nm AgNP aggregates, respectively, whereas the AEF values at 10^−8^ M RhB available for the 86- and 151-nm aggregates were 3.4 × 10^4^ and 2.2 × 10^4^. These results demonstrate that the 86-nm AgNP aggregate was the most sensitive nanostructure in the present study. The high sensitivity attained can be attributed to the optimal AgNP aggregate size, as discussed above.

### Premixed atomization of AgNPs and probe molecules

**Figure 6:**
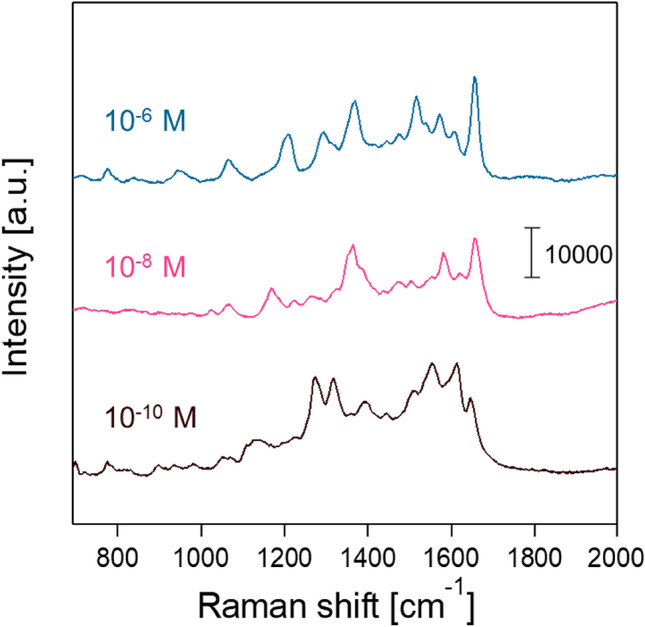
SERS spectra of the 86 nm AgNP aggregates with (**a**) 10^−6^ M, (**b**) 10^−8^ M, and (**c**) 10^−10^ M RhB in the premixed atomization route.

**Figure 7:**
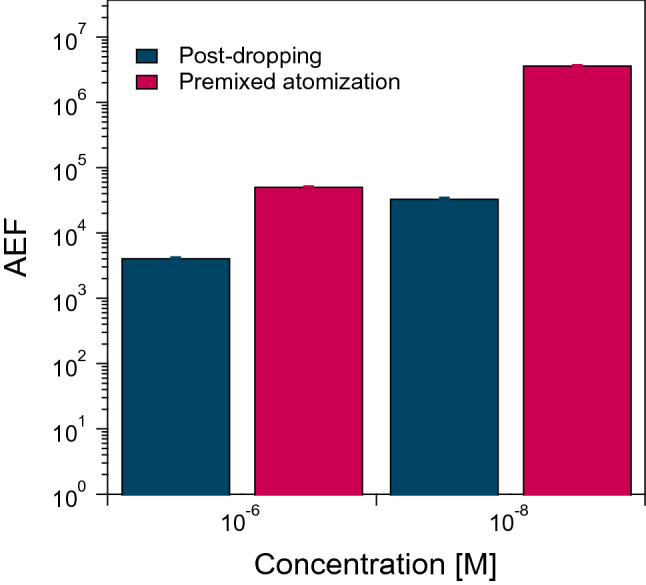
AEF values obtained from the 86 nm AgNP aggregates in the post-dropping and premixed atomization routes.

**Figure 8:**
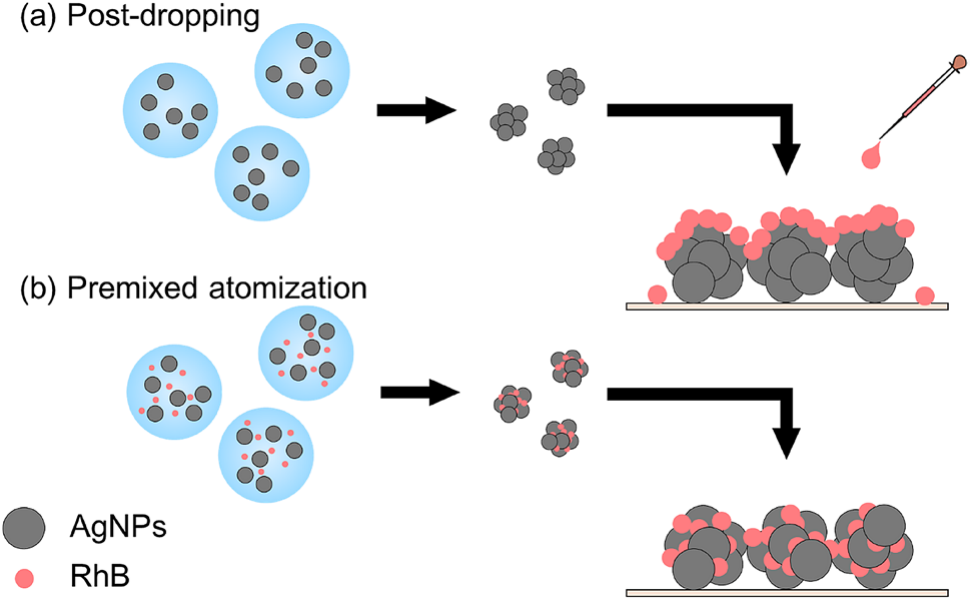
(**a**) Post-dropping and (**b**) premixed atomization routes for comparison of the hot spots they create.

Figures 4 and 5 demonstrate that the 86 nm AgNP aggregate was the most sensitive nanostructure in the SERS sensing. Here, we further explored Raman enhancement in the 86-nm AgNP aggregates by premixing RhB with the AgNP suspension before the atomization (i.e., by taking the premixed atomization route). Figure 6 shows SERS spectra of RhB at 10^−6^, 10^−8^, and 10^−10^ M concentrations in the 86-nm AgNP aggregates. Similar to the earlier experiments, no clear RhB peaks were seen at 10^–10^ M. The AEF values at 10^−6^ and 10^−8^ M RhB were found to be higher with the premixed atomization than with the post-dropping (Fig. 7). The AEF values with the premixed atomization were estimated to be 5.1 × 10^4^ and 3.7 × 10^6^ for the 10^−6^ and 10^−8^ M concentrations, respectively, or 12 and 110 times higher than the AEF values with the post-dropping. The further Raman enhancement in the premixed atomization route was attributable to the greater number of opportunities for the trapping of the probe molecules (RhB) in hot spots (Fig. 8). The increased trapping of RhB in hot spots increased AEF values.

## Conclusions

We have reported a spray-drying method to fabricate highly sensitive SERS platforms based on AgNP aerosol aggregates. AgNP aggregates of sizes, 48, 86, 151, and 218 nm, were prepared using a custom-built system. The AEFs of the 48-, 86-, 151-, and 218-nm AgNP aggregates treated by the post-dropped 10^−6^ M RhB were 2.4 × 10^3^, 4.2 × 10^3^, 3.3 × 10^3^, and 4.0 × 10^3^, respectively, whereas those treated by the post-dropped 10^−8^ M RhB, which could be detected for only the 86- and 151-nm aggregates, were 3.4 × 10^4^ and 2.2 × 10^4^. We found that the 86-nm AgNP aggregate was the most sensitive nanostructure in the present study. The higher Raman enhancement of the 86-nm aggregate versus that of the 218-nm aggregate was attributable to the deformation and damping of the electron cloud in the highly aggregated AgNPs, a phenomenon that reduced the sensitivity for Raman enhancement.

When RhB was premixed with the AgNP suspension before atomization, the Raman enhancement was greatly enhanced compared to that in the earlier experiments adopting the post-dropping route. The AEFs of the 86-nm aggregates formed by the premixed atomization route were 5.1 × 10^4^ and 3.7 × 10^6^ at the 10^−6^ and 10^−8^ M RhB concentrations, respectively. These AEFs were 12 and 110 times higher than those formed by the post-dropping route. The greater Raman enhancement in the premixed atomization route can be ascribed to the greater number of probe molecules (RhB) to be trapped in the hot spots (Fig. 8). This route is expected to be a promising method for the trace analysis of environmental pollutants.

## Methods

### Materials

Silver nanoparticles (AgNPs) were used as an SERS agent in this study. An AgNP colloidal suspension with a primary particle size of 30 nm (Ag Nanocolloid H-1, Mitsubishi Materials Corporation) was purchased and diluted with ultrapure water (18.2 MΩ cm) to obtain 0.01 and 0.1 wt% AgNP aqueous suspensions. Rhodamine B (RhB; Kanto Chemical Co., Ltd) was chosen as a probe molecule. Briefly, 47.9 mg of RhB was dissolved in 10 mL of ultrapure water to prepare a 1.0 × 10^−2^ M RhB stock solution. The RhB stock solution was further diluted with ultrapure water to obtain 1.0 × 10^−6^, 1.0 × 10^−8^, and 1.0 × 10^−10^ M RhB solutions for the SERS experiments. All materials were used without further purification.

### Atomization of the AgNPs

Atomized droplets containing AgNPs were used to fabricate AgNP aggregates for SERS sensing. A schematic of the experimental setup is shown in Fig. 1. The droplet atomization was carried out using a custom-built system with a pressurized two-fluid nozzle^49^. Briefly, a liquid sample was drawn into the system by the negative pressure occurring within the nozzle when a pressurized gas was introduced. A liquid film formed within the nozzle and was then broken into droplets through the force of the shear stress. An AgNP suspension at a given concentration was supplied to the system at a rate of 0.25 mL min^−1^ using a syringe pump (70–2205, Harvard Apparatus). Ultrapure water was mixed with the AgNPs suspension before the atomization to further dilute any concentrations of potential contaminants within the suspension. N_2_ gas was used both as a pressurized gas and as a carrier gas to deliver the atomized droplets from the nozzle to a heating zone. N_2_ gas was further introduced into the heating zone at a flow rate of 4.5 L min^−1^, and the atomized droplets were heated to 150° C to promote solvent evaporation and dry the AgNP aerosols. The total flow rate in the atomization system was 6.9 L min^–1^, and the droplet residence time was 0.93 s. Finally, AgNPs in the form of dried aerosol were deposited onto a copper-made circular substrate (SEM plate Okenshoji Co.) of 14 mm in diameter using a custom-made impactor. The diameter of the impaction nozzle was 1 mm, hence the AgNPs were accelerated at a flow rate of 17 L min^−1^ for impaction. The deposited AgNPs were kept at ambient temperature and pressure for 24 h before the SERS experiments.

AgNPs of four different aggregate sizes were fabricated, and their sensitivity in SERS analysis was examined. The smallest and largest AgNPs aggregate sizes were achieved with the 0.01 and 0.1 wt.% AgNP suspensions, respectively. The concentration played a role in controlling the number of AgNPs incorporated in a single droplet. In addition, AgNPs of medium aggregate sizes were prepared by size selection using a differential mobility analyzer. Thus, aggregates of three sizes, namely, 48, 86, 151, and 218 nm, were prepared for analysis in the SERS experiments.

### Particle characterization

The absorption spectra of the AgNP suspensions were measured using a UV–Vis spectrometer (V-550, JASCO) to confirm the plasmon resonance of the AgNP. The scanning speed and range were 200 nm min^−1^ and 200–800 nm, respectively. Note that concentration of the AgNPs suspension used in the absorption measurement, 0.001 wt%, was markedly lower than the concentration set in the experiments, as suspensions with concentrations higher than 0.001 wt% showed saturated absorption. (Fig. S2 in SI).

The hydrodynamic diameter of the AgNPs suspension was characterized using dynamic light scattering analysis (nano Partica SZ-100-Z, HORIBA). A 0.001 wt% AgNP suspension was used for the size characterizations, because multiple scattering reduced the accuracy of the measurements taken with the higher-concentration suspensions. The measurement took 120 s and was repeated three times (Fig. S3).

The particle sizes and morphologies of the AgNP aerosols generated from the system were characterized using online and offline measurements. The particle size distributions were measured using a differential mobility analyzer (DMA; 3081, TSI) coupled with a condensation particle counter (CPC; 3775, TSI) as a scanning mobility particle sizer^50^. The DMA was also used for size selection to obtain the medium-size AgNPs aggregates. The average size of the AgNP aerosols reported in this study was the peak size of the particle size distribution. To calculate the average size, the size distribution was fitted with a log-normal distribution^51–53^.

The particle sizes and morphologies of the AgNPs deposited on the substrate were characterized with a field emission scanning electron microscope (FE-SEM; JSM-7100F, JEOL). The SEM images were taken at an accelerating voltage of 15 kV. The particle size distribution the of AgNPs deposited on the copper substrate was estimated from the Feret diameter of 100 AgNPs. The particles were deposited on the substrate over an area of about 0.785 mm^2^. The deposition time was 21 s, 227 s, 126 s and 2.5 s for the 48-, 86-, 151- and 218-nm AgNP aggregates, respectively.

We examined the reproducibility of AgNP aggregate fabrication by performing triplicate independent experiments. Similar to the preparation of 86-nm AgNP aggregates, DMA was used to prepare ~ 100-nm AgNP aggregates. Figure S4 shows the SEM images of AgNPs deposited on three copper plates and their size distributions. The average aggregate size from the triplicate experiments was found to be 103 ± 8 nm (mean ± 1SD). Furthermore, the stability of AgNP aggregates was investigated. Figure S5 exhibits SEM images of and the size distributions of AgNP aggregates as a function of time since AgNP preparations. The average aggregate sizes were 97 and 95 nm after 24 and 72 h of aggregate fabrication, respectively, and the morphologies between 97- and 95-nm aggregates were comparable. These results imply that fabricated AgNP aggregates remain stable for at least 3 days. Our SERS analysis was conducted within 3 days of AgNP preparations.

### SERS analysis

The SERS experiments were performed with AgNPs aggregates of four different sizes and RhB. One hundred µL of RhB aqueous standard solution at a given concentration was dropped onto AgNP aggregates that had been fabricated on the copper substrate. The SERS spectra were obtained with a Raman spectrometer (Nanofinder HE, Tokyo Instruments) calibrated against a silicon wafer peak at 520 cm^−1^. A 532-nm laser of 0.157 mW was applied for an exposure time of 1 s. More than 10 SERS spectra at different locations were obtained for each sample. The obtained spectra were analyzed with a data processing software application (Igor Pro, WaveMetrics) for background subtraction and calculation of a peak intensity of a representative vibrational mode of RhB, 1649 cm^−1^.

## Supplementary Information

Supplementary Information.
