# Supplementary material for: Development of spray-drying-based surface-enhanced Raman spectroscopy
Source: Sci Rep. 2022 Mar 16;12:4511. doi: 10.1038/s41598-022-08598-y (PMC8927375; doi:10.1038/s41598-022-08598-y)
Supplement: Supplementary file 1 — Supplementary Information. [file 41598_2022_8598_MOESM1_ESM.pdf]

Supplementary information to Development of spray-drying-based surface-enhanced Raman spectroscopy

Chigusa Matsumoto,[1] Masao Gen,[2]* Atsushi Matsuki,[3] Takafumi Seto[1]*

1) Graduate School of Science and Technology, Kanazawa University, Kanazawa 920-1192, Japan
2) Faculty of Frontier Engineering, Institute of Science and Engineering, Kanazawa University, Kanazawa 920-1192, Japan
3) Institute of Nature and Environmental Technology, Kanazawa University, Kanazawa 920-1192, Japan

*Author to whom correspondence should be addressed

Email: mgen@staff.kanazawa-u.ac.jp; t.seto@staff.kanazawa-u.ac.jp

Text S1. The average droplet diameter

The number concentration of the colloidal suspension of AgNPs (N_{AgNPs}) 0.01 wt% is 4.3×10^{11} $\mathrm{mL^{-1}}$. At this concentration, it is assumed that it is dispersed in the droplet and 1 AgNP is present per droplet. The number of particles in the droplet (n) can be represented by Eq (S1) using the droplet diameter (D_{d}) and the number concentration of the colloidal suspension (N_{AgNPs}).

$$n = \frac{\pi D_{\mathrm{d}}^{3}}{6} \times N_{\mathrm{AgNPs}} \qquad \text{(S1)}$$

The average droplet diameter can be calculated by calculating D_{d} at which $n = 1$.

Text S2. Primary particle size of AgNPs

Figure S6 exhibits the SEM image obtained by dropping the AgNPs colloid suspension on a copper plate and then drying it. The average primary particle size was measured to be 30 nm.

Text S3. Bouncing of particles may redistribute the size distributions of AgNPs deposited on the substrate.

Here we consider the difference in particle size distribution between SEM and SMPS from the Stokes number.
Function $C_{\mathrm{c}}(d_{\mathrm{s}})$, for Cunningham coefficient for Stokes diameter d_{s}
Stokes number of the impactor is expressed as

$$Stk = \frac{C_{\mathrm{c}}(d_{\mathrm{s}})\rho_{\mathrm{P}} d_{\mathrm{s}}^{2} U}{9\mu W} \qquad \text{(S2)}$$

where ρ_{P} is the AgNPs density, U is the average flow velocity at the nozzle, μ is the gas viscosity, W is the nozzle diameter. Stokes number of 48, 86, 151 and 218nm AgNPs are 3.18, 5.74, 10.2 and 14.8. The collection efficiency is almost 100% for any particle size, and they can be collected on the substrate. However, these Stokes numbers are much larger than the Stokes values at a collection efficiency of 50%, and the probability that particles colliding with the substance will bounce back without being collected. The velocity of particles (U_{i}) when they collide with the collision plate is estimated from the

empirical formula of rebound of particles[1].

$$\frac{U_{\mathrm{i}}}{U} = 1 - \frac{1}{4Stk} + \frac{1}{96Stk^2} \tag{S3}$$

The conditions under which particle rebound is predicted are as follows.

$$U_{\mathrm{i}} d_s \sqrt{\frac{\rho_{\mathrm{P}}}{\rho_0}} > \Gamma \times 10^{-6}\ m^2 \cdot s^{-1} \tag{S4}$$

where ρ_{P}/ρ_0 obtained by dividing the particle density [gcm^{-3}] and, Γ has values ranging from 2.5 to 9.2 depending on the material and size of particles, type of impactor, etc. As a general criterion for particle bounce in impactors, a value of 5 is chosen for Γ. Value on the left side of Eq (S4) of 48, 86, 151 and 218 nm AgNPs are 1.41 × 10^{-5}, 2.62 × 10^{-5}, 4.43 × 10^{-5} and 6.76 × 10^{-5}. Any of the particle sizes satisfies Eq (S4) and the particles are expected to rebound. It suggests that the bouncing of the particles altered the particle size distribution on the substrate and affects the difference from the SMPS distribution.

Text S4. The UV-Vis spectrum and the size distribution of AgNPs in the suspention

The UV-Vis spectrum of AgNP suspension shows an absorption peak at around 395 nm (Fig. S2). The peak corresponds to the localized surface plasmon resonance (SPR) of AgNPs[2] that initiates SERS. Figure S3 shows the size distribution of AgNPs in the suspension of 0.001 wt% measured by DLS analysis. The average size of AgNPs was found to be about 46 nm that is roughly consistent with the primary particle size of AgNP (30 nm) and the average size of AgNP aerosols (38 nm).

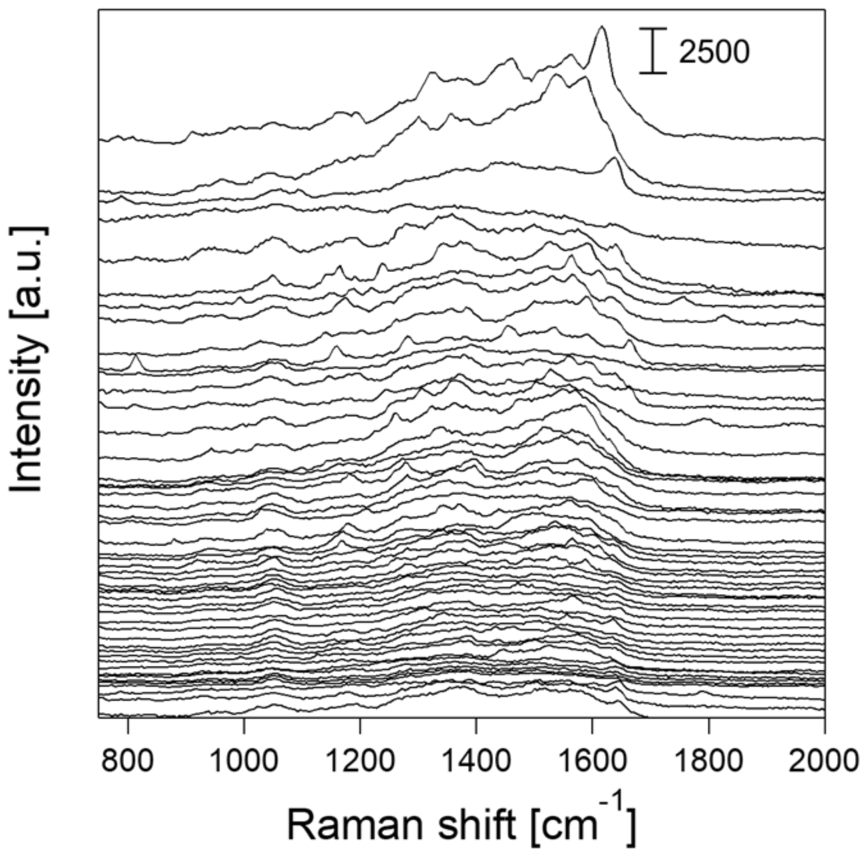

Figure S1. SERS spectra of RhB at the 10^{-6} M concentration with the colloidal suspension method.

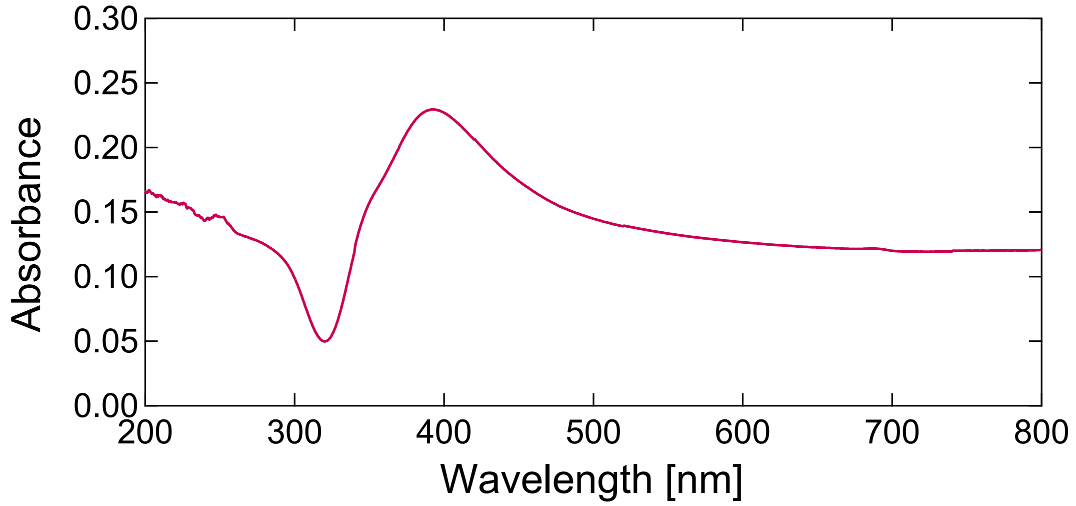

Figure S2. The UV-Vis spectrum of AgNP suspension

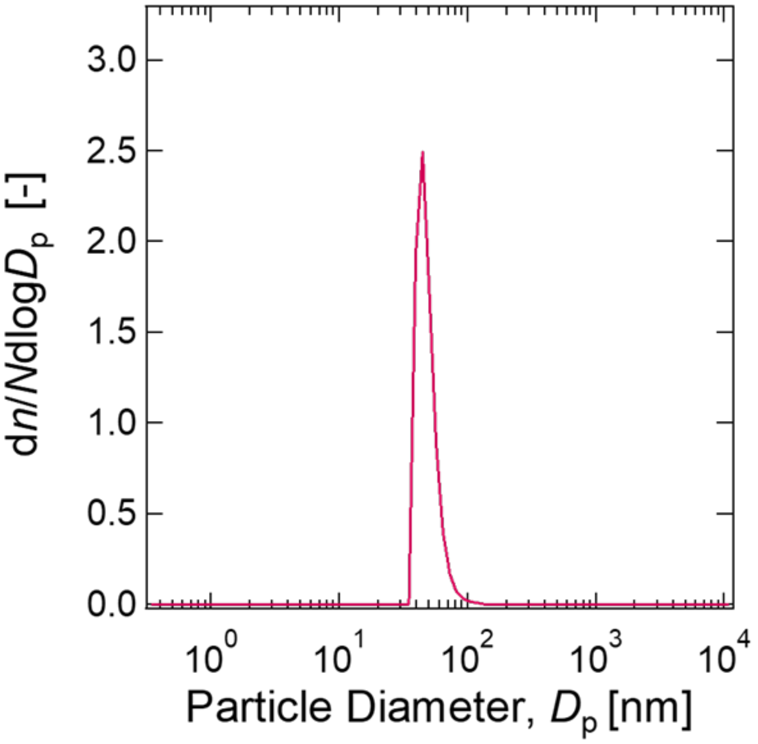

Figure S3. the size distribution of AgNPs in the suspension of 0.001 wt% measured by DLS analysis

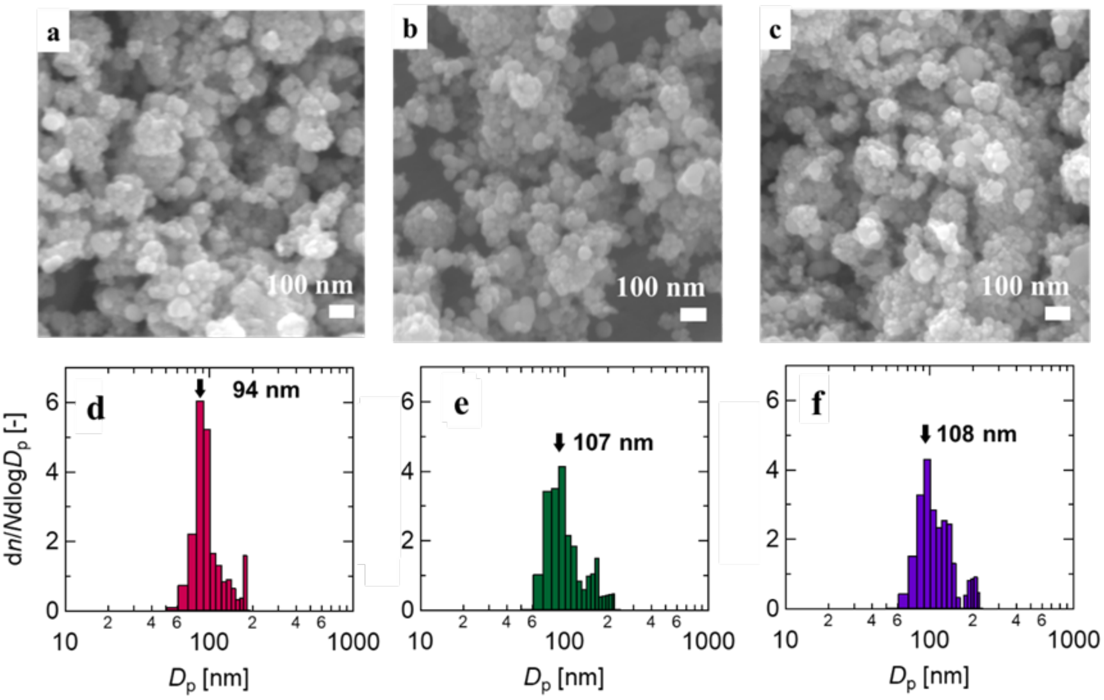

Figure S4. SEM images and size distributions of the deposited AgNPs. (a, d) sample 1, (b, e) sample 2, and (c, f) sample 3.

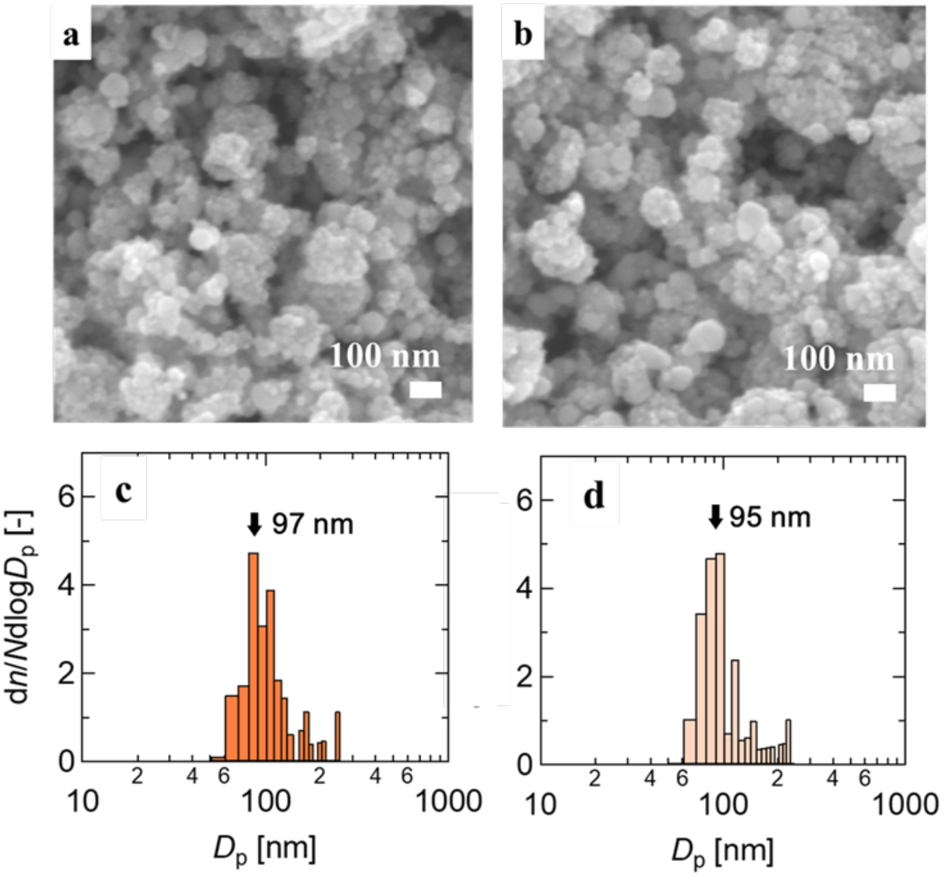

Figure S5. SEM images and size distributions of deposited AgNPs after (a, c) 24 and (b, d) 72 hours of aggregate fabrication.

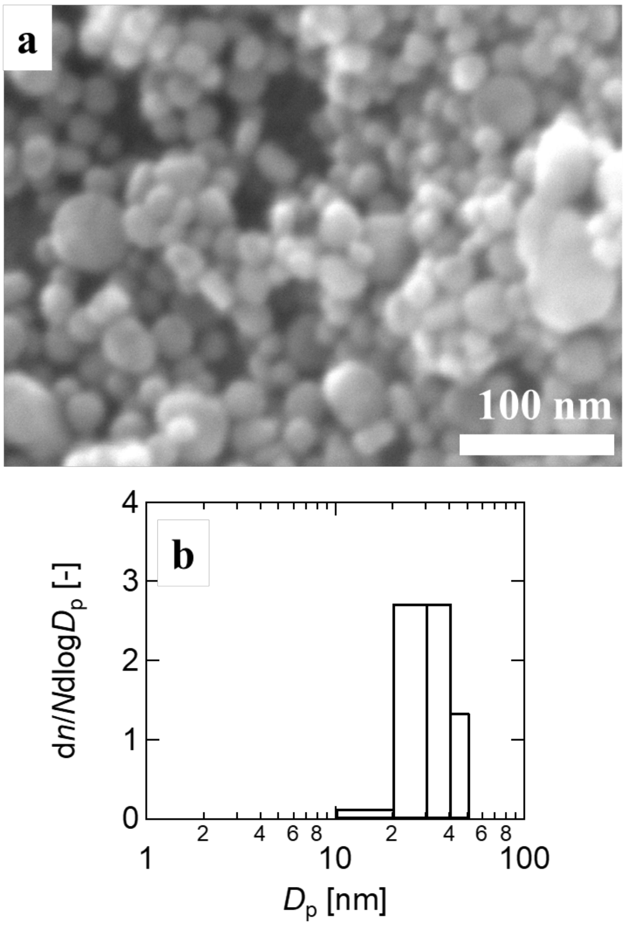

Figure S6. (a) SEM images and (b) size distributions of deposited AgNPs

References

1. Cheng, Y. S. & Yeh, H. C. Particle Bounce in Cascade Impactors. *Environ. Sci. Technol.* **13**, 1392–1396 (1979).

2. Dutra, M. A. L., Marques, N. do N., Fernandes, R. da S., de Souza Filho, M. de S. M. & Balaban, R. de C. ECO-FRIENDLY hybrid hydrogels for detection of phenolic RESIDUES in water using SERS. *Ecotoxicol. Environ. Saf.* **200**, 110771 (2020).
